# Laparoscopic splenectomy: a new approach

**DOI:** 10.6061/clinics/2018/e16-536

**Published:** 2018-11-16

**Authors:** Qian-jin He, Xiao-meng Dai, Chao Yu, Sheng-li Yang

**Affiliations:** I Department of General Surgery, Huanggang Central Hospital, Hubei 438000, P.R. China.; II Cancer Center, Union Hospital, Tongji Medical College, Huazhong University of Science and Technology, Wuhan 430022, China.; III Department of Hepatobiliary Surgery, Affiliated Hospital of Guizhou Medical University, Guiyang, Guizhou 550001, P.R. China

**Keywords:** Laparoscopic Splenectomy, Approach, Pancreatic Tail

## Abstract

**OBJECTIVES:**

To introduce a new laparoscopic splenectomy (LS) approach.

**METHODS:**

Sixteen patients underwent LS with general anaesthesia and carbon dioxide pneumoperitoneum. The details of the surgery are as follows: 1. The omentum was incised along the greater curvature and retracted as much as possible to expose the pancreatic body and tail. 2. The right arteriovenous root in the gastric omentum was ligated to sufficiently expose the pancreatic body and tail. 3. The pancreatic capsula was opened along the inferior margin of the pancreatic tail, elevated and separated until the superior margin of the pancreas was grasped. The entire splenic pedicle was retracted using a string. The branching blood vessels in the splenic hilus were ligated using clamps and separated. The splenogastric and splenophrenic ligaments were transected proximally using an ultrasonic knife, and the thick short gastric blood vessels were clamped. This procedure allows complete exposure of the area above the pancreatic tail where the splenic hilus is located. The splenoportal vasculature was suspended using a 7–0 silk suture to easily manipulate this tissue. The splenic portal vessels were dissected using an ultrasonic knife, and the portal vessels were isolated individually using vascular clamps and transected. The splenogastric and lienorenal ligaments were also transected. The spleen was then placed into a bag, and the surgical port was slightly enlarged. Finally, the spleen was sectioned for removal.

**RESULTS:**

Fifteen surgeries were successfully performed from March 2015 to January 2016. One patient underwent laparotomy. No patients developed postoperative intra-abdominal haemorrhage or infection. One patient developed subcutaneous emphysema, and one developed a wound infection. No deaths occurred.

**CONCLUSIONS:**

Active exposure of the area dorsal to the pancreatic tail is a safe and simple splenectomy method.

## INTRODUCTION

Laparoscopic splenectomy (LS) has been widely performed since the technique was first reported in 1991 1; it is indicated as a method for splenectomy following laparoscopic cholecystectomy (LC) 2,3. LS was initially a highly difficult surgery that required experienced, skilled physicians and advanced medical equipment. One slight mistake may cause massive haemorrhage with a high risk of conversion to open surgery 4. Different operative approaches of varying degrees of complexity have been developed 5,6. We recently established a method of manipulating the splenic hilum via active exposure of the pancreatic tail.

## MATERIALS AND METHODS

Sixteen patients underwent this approach from March 2015 to January 2016, including six males and ten females with an average age of 36 years (15–63 years). The preoperative diagnoses were idiopathic thrombocytopenic purpura (ITP) in nine patients, hypersplenism secondary to liver cirrhosis due to hepatitis B in three patients, splenic cysts (large hydatid cysts, which typically induce symptoms due to compression of the peripheral organs) in two patients, splenic lymphatic vessel tumours in two patients, and splenic haemangiomas (space-occupying lesions that cannot be preoperatively diagnosed as benign lesions) in three patients. Nine patients exhibited no splenic enlargement, nine patients exhibited mild-to-moderate splenic enlargement, and four patients exhibited severe splenic swelling. Indwelling nasogastric tubes were placed in all patients, and preoperative prophylactic antibiotics were administered to all patients. ITP patients also received appropriate treatments. The Institutional Review Board at the Center Hospital of Huanggang approved the study, and all patients voluntarily provided written informed consent prior to surgery.

### Surgical methods

**Figure 1:**
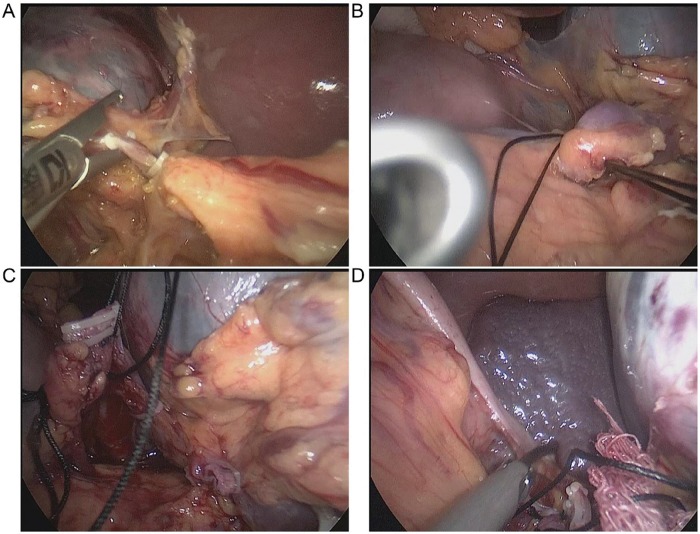
Intraoperative images of the new laparoscopic splenectomy method. **A:** Dissection and incision of the left gastroepiploic artery and vein. **B:** Splenic hilum suspended using a 7–0 silk suture. **C:** Dissection and ligation of the splenic hilar vessels. **D:** Ligation of the short gastric artery and vein.

All patients received general anaesthesia and carbon dioxide pneumoperitoneum (pressure: 13 mmHg) with tracheal intubation in a supine position with the head elevated. The surgeon stood on the right side of the patient, and a 10-mm trocar was inserted through an observation port that was created via an incision just below the umbilicus. Another 10-mm trocar was introduced through the main port, which was located just below the left costal margin. A 5-mm incision was used to create an auxiliary port at the midpoint between the xiphisternum and umbilicus. We opened the gastrocolic ligament using an ultrasound knife, entered the omental bursa, and transected the splenocolic ligament. We dissected and transected the left gastroepiploic artery and vein (Figure 1A) and separated any adhesions between the greater omentum and spleen. We opened the surface capsule surrounding the pancreatic tail and dissected proximally while visualizing the branch of the pancreatic tail vessels composed of the splenic artery and vein and then transected these vessels. This complete sequence was performed to fully expose the pancreatic tail, where the splenic hilum is located in the upper portion. The splenic hilum was suspended using 7–0 silk suture (Figure 1B). The splenic hilum was easily manipulated to visualize and ligate the splenic hilar vessels using an ultrasonic scalpel, and each vessel was clamped using a vascular clip (Figure 1C). The use of a vascular closure device simplified the surgery. The gastrosplenic and splenorenal ligaments were transected using an ultrasonic knife (Figure 1D). The spleen was placed into a bag, and the main surgical port was enlarged. The spleen was sectioned into small pieces for removal, a drainage tube was routinely placed in the splenic fossa.

## RESULTS

Fifteen of the 16 surgeries were successful. One patient with a severely enlarged spleen underwent conversion to open surgery because of rapid haemorrhage due to failure to expose the pancreatic tail. The mean operative time was 120 min (75–160 min), and the total intraoperative blood loss was 50–600 mL. One patient developed subcutaneous emphysema that did not require special treatment, and one patient developed infection of the main surgical incision but completely recovered following dressing changes. No postoperative haemorrhage, abdominal infections, pancreatic fistulas, other complications or death occurred. Pancreatitis is most often diagnosed by the presence of two of the following three criteria: 1) abdominal pain consistent with the disease, 2) serum amylase and/or lipase concentrations greater than three times the upper limit of normal, and/or 3) characteristic findings on abdominal imaging (strong recommendation, moderate quality of evidence) 7. A pancreatic fistula was defined according to the International Study Group of Pancreatic Fistula (ISGPF) criteria as any measurable drainage from a surgically placed drain (or a subsequently placed percutaneous drain) on or after postoperative day 3 with a serum amylase content greater than 3 times the upper limit of normal (300 IU/L). All patients with values below this threshold were diagnosed with pancreatitis and/or a pancreatic fistula 8.

## DISCUSSION

LS causes less trauma and ensures a faster recovery than open splenectomy, and it is a safe and effective method 9-12. However, the spleen sits in a deep position and exhibits has a friable consistency, and the pancreatic tail is located near the spleen. Therefore, LS is a highly risky, difficult, and challenging surgery for most surgeons.

Initial separation of the splenic artery is suggested for most conventional LS procedures to avoid massive haemorrhage during surgery, followed by transection of the splenocolic ligament, opening of the lienorenal ligament, and ligation of the splenic artery branches near the surface of the spleen. Endo-GIA or LigaSure may be used for this process 6. This surgical approach has a higher risk of haemorrhage, especially in patients with hypersplenism and portal hypertension because many congestive vascular branches are located in the splenorenal ligament. Endoscopic haemostasis is extremely difficult to achieve once haemorrhage occurs, and it is accompanied by the risk of injury to the pancreatic tail.

Separation of the splenic artery is not necessary at the beginning of surgery if the splenic hilum is manipulated via active exposure of the pancreatic tail. We entered the omental bursa and removed adhesions of the greater omentum and spleen. We ligated and transected the left gastroepiploic artery and vein, placed the left side of the greater omentum in the lower abdomen, opened the pancreatic tail capsula from its margo inferior, and dissected dorsally to where fewer blood vessels are present. Splenic tissue is present dorsal to the stomach. We suspended the splenic hilum using 7–0 silk suture to control the splenic pedicle. This technique caused little haemorrhage and was associated with less injury to the pancreatic tail than other techniques. The peritoneum was excised proximal to the lienorenal ligament, and the lienophrenic and splenogastric ligaments were transected dorsally. This method is difficult to perform when a severely enlarged spleen completely covers the pancreatic tail because this limited abdominal space cannot be exposed. One patient with such a severely enlarged spleen underwent conversion to open surgery, but the conventional approach was also very difficult.

The pancreatic tail was exposed during surgery, and the entire splenic pedicle was able to be controlled. Visualization was clear because of negligible haemorrhage during the surgery, which greatly reduced the incidence of injury or trauma to the pancreatic tail and led to faster recovery among these patients than among those undergoing other procedures. In conclusion, active exposure of the pancreatic tail is a safe and effective method for controlling the splenic hilum during LS. However, no clinical comparisons with other surgical methods were performed because of the limited number of patients included in this study, and more comprehensive investigations are necessary in the future.

## AUTHOR CONTRIBUTIONS

Yang SL, He QJ and Dai XM designed the study. He QJ, Dai XM and Yu C collected and analyzed the data. He QJ and Dai XM wrote the manuscript. All authors read and approved the final version of the manuscript.
